# Fragmentation of Nuclear Remnants in Electron–Nucleus Collisions at High Energy as a Nonextensive Process

**DOI:** 10.3390/e28040470

**Published:** 2026-04-20

**Authors:** Ting-Ting Duan, Sahanaa Büriechin, Hai-Ling Lao, Fu-Hu Liu, Khusniddin K. Olimov

**Affiliations:** 1 State Key Laboratory of Quantum Optics Technologies and Devices, Institute of Theoretical Physics, Shanxi University, Taiyuan 030006, China; 202312602001@email.sxu.edu.cn (T.-T.D.); 202201101236@email.sxu.edu.cn (S.B.); 2 Collaborative Innovation Center of Extreme Optics, Shanxi University, Taiyuan 030006, China; 3 Department of Science Teaching, Beijing Vocational College of Agriculture, Beijing 102442, China; hailinglao@pku.edu.cn; 4 Laboratory of High Energy Physics, Physical-Technical Institute of Uzbekistan Academy of Sciences, Chingiz Aytmatov Str. 2b, Tashkent 100084, Uzbekistan; 5 Department of Natural Sciences, National University of Science and Technology MISIS (NUST MISIS), Almalyk Branch, Almalyk 110105, Uzbekistan

**Keywords:** *α*-cluster structure, nuclear structure, multiplicity distribution of nuclear fragments, nonextensive process, the Electron-Ion Collider, 21.60.Gx, 21.60.-n, 21.60.Ka

## Abstract

Utilizing a partitioning method based on equal (or unequal) probabilities—without incorporating the alpha-cluster (*α*-cluster) model—allows for the derivation of diverse topological configurations of nuclear fragments resulting from fragmentation. Subsequently, we predict the multiplicity distribution of nuclear fragments for specific excited nuclei, such as Be*9, C*12, and O*16, which can be formed as nuclear remnants in electron–nucleus (eA) collisions at high energy. Based on the α-cluster model, an α-cluster structure may result in deviations in the multiplicity distributions of nuclear fragments with charge Z=2, compared to those predicted by the partitioning methods. Furthermore, in the framework of Tsallis statistics, the nonextensive generalized temperature, entropy index, and *q*-entropy are obtained from the multiplicity distribution of nuclear fragments with a given charge number. Our work shows that fragmentation of nuclear remnants in electron–nucleus collisions at high energy is a nonextensive process.

## 1. Introduction

High-energy nuclear collisions represent a significant area of research within modern physics [1,2,3,4,5]. In these interactions, numerous particles are predominantly generated within participant regions while multiple fragments are primarily emitted from spectator regions when available. A series of recent research achievements focusing on the field of nuclear fragmentation—spanning model construction, experimental planning, and mechanism analysis—comprehensively demonstrate the vigorous development trend of this field, providing multiple perspectives for a deeper understanding of nuclear structure and nuclear reaction dynamics [6,7,8,9,10,11,12].

Relevant research content includes, but is not limited to, the phenomenon of nuclear fragmentation in the intermediate energy region, the influence of nuclear collective excitation modes on nuclear fragmentation, the competition between nuclear fragmentation and electromagnetic fragmentation, how nuclear micro-excited states affect the production of macroscopic nuclear fragments, and the application of Monte Carlo methods in nuclear fragmentation studies [6,7,8,9,10,11,12]. These studies complement and advance one another, not only propelling the field of nuclear fragmentation forward but also laying a solid foundation for progress in related areas such as nuclear physics and nuclear technology applications.

During multi-fragment emission processes, it is anticipated that spectators will form an excited nucleus. This excited nucleus subsequently undergoes fragmentation into various components [13,14,15,16]. The fragmentation reveals rich internal structures within the nucleus, where protons and neutrons can combine appropriately to create intermediate configurations [17,18,19,20]. When two protons and two neutrons coalesce into such an intermediate structure, it is referred to as an alpha-cluster (α-cluster) structure [21,22,23,24]. It is possible that two or more α-cluster structures are existent in a heavy nucleus [25,26,27,28,29]. According to the α-cluster model [30,31,32,33,34], the α-cluster structure should have much higher probability than other intermediate structures.

Different configurations of nuclear fragments can be measured in the fragmentation of excited nuclei. A multi-α configuration is one such arrangement that arises from statistical or stochastic fragmentation processes. Several experimental results concerning multi-*α* configurations in O16 fragmentation at high energies have been reported in the literature [13,35,36,37,38]. According to the α-cluster model [30,31,32,33,34], further evidence and a higher probability of the presence of α-cluster structures are anticipated. If the occurrence of multi-α configurations is significantly more probable than what would be expected based on partitioning methods derived from stochastic processes, it may indeed reflect the underlying α-cluster structure of the excited nucleus.

Due to challenges in excluding the influence of partitioning probabilities across various configurations, there remains limited experimental evidence supporting α-cluster structures; this evidence is insufficient for a comprehensive validation of the α-cluster model [30,31,32,33,34]. Consequently, there is a pressing need for more systematic experimental investigations. This necessity motivates researchers to measure fragmentation products originating from excited nuclei. It is anticipated that diverse types of excited nuclei (nuclear remnants) will be formed through nuclear reactions induced by electrons at the forthcoming Electron-Ion Collider (EIC) [39]. The EIC presents an exceptional opportunity for researchers to systematically explore α-cluster structures and thoroughly validate the α-cluster model using fragmentation products from excited nuclei.

Furthermore, nuclear fragmentation is a non-thermal equilibrium process, rendering Boltzmann–Gibbs statistics inapplicable. Instead, one may adopt two-, three-, or multi-component distributions, where each component represents a state of local equilibrium that can be described within the framework of Boltzmann–Gibbs statistics. Consequently, a multi-temperature pattern, or temperature fluctuations, can be observed in nuclear fragmentation processes. Empirically, such multi-component distributions can be effectively fitted using Tsallis statistics, which indicates that nuclear fragmentation is inherently a nonextensive process. Therefore, nonextensive parameters derived from Tsallis statistics, including generalized temperature, entropy index, and *q*-entropy, can be employed to characterize and analyze nuclear fragmentation phenomena.

In this work, both equal and unequal probability partitioning methods are employed to derive various configurations of nuclear fragments from excited states such as Be*9, C*12, and O*16 which are expected to result from electron–nucleus (eA) collisions at the EIC [39]. The multiplicity distributions for all fragments, as well as those with charge *Z*, will subsequently be obtained. In particular, the probability of a multi-α or multi-He configuration or channel can be obtained, which may serve as the baseline for judging about the α-cluster structure. In addition, in the framework of Tsallis statistics, the nonextensive generalized temperature, entropy index, and *q*-entropy are obtained from the multiplicity distribution of nuclear fragments with a given charge number.

The remainder of this paper is structured as follows. Various configurations of nuclear fragments are described in Section 2. Multiplicity distributions of nuclear fragments are presented in Section 3. In Section 4, we show nonextensive parameters from multiplicity distributions of nuclear fragments. Finally, we provide a summary and the conclusion of this work in Section 5.

## 2. Various Configurations of Nuclear Fragments

In the context of multi-fragment emission during eA collisions, various fragmentation properties warrant special attention. For instance, understanding the types of fragments is crucial for elucidating the mechanisms underlying nuclear fragmentation; however, determining the number of neutrons in an isotope presents a complex challenge. Our previous research has demonstrated that the isotopic production cross-section follows an Erlang distribution [40]. When different isotopes with a given charge number are not distinguished from one another, the analysis becomes significantly more straightforward.

At the EIC, as nuclear remnants, excited nuclei formed in eA collisions can fragment into diverse topological configurations. This allows for an investigation into the internal structure of these excited nuclei. During fragmentation, both proton and neutron numbers are conserved. In experimental settings, it is possible to measure either the charge or proton count of a fragment. This capability facilitates our examination of multiplicity distributions among fragments with varying charges. Furthermore, we may delve deeper into discussing the fundamental physical reasons behind these multiplicity distributions observed in nuclear fragments. Notably, factors such as α-cluster structures and liquid–gas phase transitions could influence these experimentally measured multiplicity distributions.

As examples, we now consider three types of eA collisions at the EIC:

(1) e+10Be⟶{(e+n)+9Be*(e+p)+Li*9, Li*9⟶2n+7Li*or3n+Li*6,

(2) e+C13⟶{(e+n)+C*12(e+p)+B*12,B*12⟶n+B*11or2n+B*10,

and

(3) e+O17⟶{(e+n)+O*16(e+p)+N*16, N*16⟶n+N*15or2n+N*14,

in which the excited Be9, C12, and O16 nuclei can be obtained and analyzed. Other excited nuclei are not the focus of the present work due to the fact that they do not have an advantage in the study of α-cluster structure.

It is important to note that the process e+n or e+p occurring in eA is not an electron-induced neutron/proton knock-out reaction, which typically manifests at beam energies of hundreds of MeV [41,42]. Instead, this represents a multi-particle production process that occurs at beam energies on the order of hundreds of GeV, which the EIC is specifically designed to achieve [43], with a center-of-mass energy range between 20 and 100 GeV. In the incident nucleus *A*, alongside the participant nucleon, there exist spectator nucleons—the remaining constituents—which will form an excited nucleus characterized by energy levels significantly higher than those attainable through MeV collisions.

The excited nuclei subsequently decay into various nuclear fragments. The correlations between momentum and scattering angle for evaporated neutrons and protons have been extensively studied using the BeAGLE (Benchmark *eA* Generator for Leptoproduction) model [44] (the version number has not been disclosed, and it is speculated to be the initial version), particularly in high-energy lepton–nucleus collisions. The excited nuclei produced in eA collisions at the EIC exhibit significantly higher excitation levels compared to those generated in electron-induced neutron/proton knock-out reactions conducted with fixed targets at low and medium energies. Due to substantial excitation leading to large internal momenta, both decay protons and other nuclear fragments correspond to sufficiently large polar angles that fall within the estimated pseudorapidity acceptance region designated for the currently proposed EIC detector [39,45].

Considering the Fermi momentum of a nucleon within the nucleus, which is approximately 0.25 GeV/c, and the momentum per nucleon of the incident nucleus being 10 GeV/c, decay protons and other fragments are expected to be emitted within a forward cone characterized by a polar angle $\theta_{0}=25$ mrad. This corresponds to a pseudorapidity of $\eta =-\ln\tan(\theta_{0}/2)=4.38$. Furthermore, recoil protons can be distinguished from decay protons since recoil protons participate in multi-scattering processes that result in much larger scattering angles. It is assumed that this emission will span a wide range from nearly 0 (corresponding to η=∞) up to approximately $10\theta_{0}$ (which corresponds to η=2.07). Indeed, it cannot be excluded that some recoil protons may have very small scattering angles, leading to exceptionally large pseudorapidities due to the influence of leading nucleons.

If the two types of protons are assumed to emit isotropically in their respective rest frames, they approximately follow Gaussian η distributions with a common standard deviation ($\sigma_{\eta }\approx 0.91$). The decay protons predominantly distribute within the range of $4.38<\eta <4.38+4\sigma_{\eta }=8.02$, while the recoil protons primarily occupy the range of $2.07<\eta <2.07+4\sigma_{\eta }=5.71$. It is evident that there exists some overlap between decay and recoil protons in the forward cone. Although most recoil protons may have emission angles exceeding 25 mrad due to multiple scattering processes, we cannot entirely dismiss the possibility of them appearing within the forward cone. To more effectively distinguish between decay and recoil protons, one could study their energies; generally, the energy of a decay proton is nearly equal to that of an incident nucleus per nucleon, whereas the energy of a recoil proton should be lower.

While it is challenging to precisely separate decay from recoil protons in the forward cone, such a distinction is not essential for this study. In fact, among the three types of eA collisions considered in previously mentioned reactions (1)–(3), our selected samples should ideally consist solely of those with only recoil neutrons; thus, any contributions from recoil protons must be excluded from our analysis. In rare instances where mixed events occur involving recoiling protons within the forward cone, these can introduce minor measurement errors. In cases where recoiling protons do appear in this region, misidentified events would include both these recoils and fragmentations from Li*9, B*12, and N*16, respectively—these should be excluded from expected events associated with fragmentations originating from Be*9, C*12, and O*16. Although there are clear differences in energy between decay and recoil protons, attempting to separate them experimentally may incur additional costs.

In our studies concerning nuclear fragments, our primary focus lies in counting electric charges rather than mass, momentum, energy, and so on. The resolutions of detectors regarding secondary quantities do not impact our analysis; however, it is crucial that the detector resolution for charge number remains high—approximately ∼2%, which is generally achievable. During experiments, it is essential to select relevant decay events where the total charge number of various nuclear fragments precisely matches that of the incident nucleus *A*. In some events, due to distribution fluctuations, individual nuclear fragments have a certain probability of emitting with a very small polar angle, which is mixed with the beam and cannot be captured by the detector [39,43,45]. Naturally, these events should be removed from the analysis.

In addition to $N_{F}$ representing the multiplicity of all nuclear fragments, let $N_{Z}$ be the multiplicity of the fragments with charge *Z*. In an equal probability partitioning method, in which the α-cluster model does not enter, the frequency of configuration $\left\{N_{Z}\left(Z\right)\right\}$ or the weight of partition $\left\{N_{Z}\left(Z\right)\right\}$ is considered to be the same, which results in the same probability $f_{1}$. Various topological configurations of nuclear fragments in excited nuclear fragmentation can be obtained by the treatment of exhaustive enumeration.

In an unequal probability partitioning method [46,47], in which the α-cluster model does not enter either, the frequency of configuration $\left\{N_{Z}\left(Z\right)\right\}$ or the weight of partition $\left\{N_{Z}\left(Z\right)\right\}$ is considered to be the number of exchange

(4) $$\begin{array}{c} M_{2}=\frac{Q!}{\prod_{Z}N_{Z}\left(Z\right)!Z^{N_{Z}}}, \end{array}$$

where *Q* is the charge number of the excited nucleus, Q! and $N_{Z}\left(Z\right)!$ represent factorial operations, and $M_{2}$ is the Cauchy number in the number theory. The normalization of $M_{2}$ is

(5) $$\begin{array}{c} \sum_{\left\{N_{Z}\left(Z\right)\right\}}M_{2}=Q! \end{array}$$

in which the probability of configuration $\left\{N_{Z}\left(Z\right)\right\}$ is

(6) $$\begin{array}{c} f_{2}=\frac{M_{2}}{\sum_{\left\{N_{Z}\left(Z\right)\right\}}M_{2}}=\frac{1}{\prod_{Z}N_{Z}\left(Z\right)!Z^{N_{Z}}}. \end{array}$$

The equal and unequal probability partitioning methods present distinct perspectives in the realm of physics. The equal probability partitioning method is grounded in the principle of equal probability, a fundamental assumption in statistical physics. This principle asserts that when a system is at equilibrium, provided there are no additional constraints beyond energy, volume, and particle number, the likelihood of the system occupying each microscopic state remains uniform. Conversely, the unequal probability partitioning method relies on the principle of unequal probability; this acknowledges that within a sampling survey, the chance of selecting any individual from a population may vary due to the interchangeability of identical particles.

Prior to implementing the partitioning methods, it is essential to highlight other applications that demonstrate their validity and rationality. In previous studies [48,49,50,51,52], these methods were employed to investigate excited nuclear fragmentation during nucleus–nucleus collisions at intermediate and high energies. Conditional moments and their normalized forms across various orders were introduced [48,49] for examining critical behavior [53,54]. It was observed that correlations and distributions derived from conditional moments of nuclear fragments [50,51,52] obtained through the partitioning technique [46,47] align well with experimental data concerning excited nuclear fragmentation resulting from diffractive excitation (nuclear reaction) as well as electromagnetic dissociation [55,56].

Using the equal (unequal) probability partitioning method, various topological configurations of nuclear fragments in excited Be9, C12, and O16 fragmentation are listed in Table 1, Table 2 and Table 3, respectively, in which each configuration has an equal (unequal) probability $f_{1}$ ($f_{2}$). The multiplicity, $N_{F}$, of all fragments and the multiplicity, $N_{Z}$, of the fragments with given charge *Z* in a defined configuration are shown separately.

In the equal probability partitioning method, the numbers of configurations, or fragmentation channels, in the fragmentation of excited B9, C12, and O16 nuclei are 5, 11, and 22, respectively. The fragment Be is artificially assumed by default with 50% probability to be the most unstable Be8 and in 50% of the cases to be (relative) stable isotope of Be. In addition, Be8 can decay into 2He, which is listed in brackets with fractions in the tables. In the unequal probability partitioning method, the numbers of exchanges in excited Be9, C12, and O16 fragmentation are 24, 720, and 40,320, respectively. The fragment Be is assumed by default with a given chance ($\left\{M_{2}\left(2\mathrm{He}\right)/\left[M_{2}\left(2\mathrm{He}\right)+M_{2}\left(\mathrm{Be}\right)\right]=1/3\right\}$) to be Be8, which is unstable and can decay into 2He with given fractions.

## 3. Multiplicity Distributions of Nuclear Fragments

After systematically deriving distinct fragmentation channels using established partitioning methods, we conduct a fragment-specific charge analysis within each channel. This involves isolating individual fragments to precisely measure and validate their charge states, leaving no detail unexamined. Simultaneously, we meticulously track two key metrics: the total fragment multiplicity per channel and the specific multiplicities of fragments with predefined target charges. By applying the corresponding weights from the partitioning methods to the multiplicity data, we accurately determine the final multiplicity distributions for both the complete fragment set and charge-specified subsets, thereby revealing the inherent probabilistic nature of nuclear fragmentation processes.

The $N_{F}$ distribution, $dn/dN_{F}$, and the $N_{Z}$ distribution, $dn/dN_{Z}$, in fragmentation of excited Be9, C16, and O16 nuclei are given in Table 4, Table 5 and Table 6, respectively, where *n* denotes the frequency of $N_{F}$ occurring. The multiplicity distributions $dn/dN_{F}$ and $dn/dN_{Z}$ are also the yield distributions of nuclear fragments. The normalization constants of $dn/dN_{F}$ and $dn/dN_{Z}$ in the partitioning methods are the numbers of configurations (exchanges).

**Figure 1:**
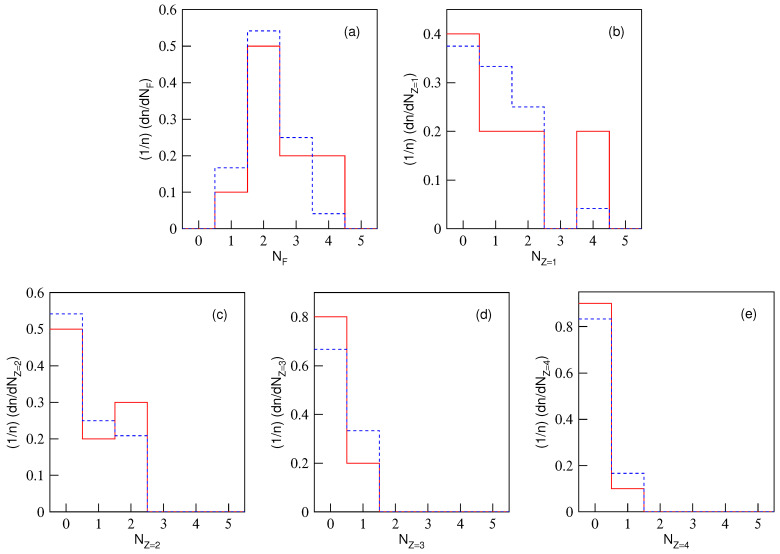
Multiplicity distributions of nuclear fragments with different charges in Be9 fragmentation. The solid (dashed) histograms represent the results from the equal (unequal) probability partitioning method. Panel (**a**) is for all fragments. Panels (**b**–**e**) are for the fragments with charge Z=1, 2, 3, and 4, respectively.

**Figure 2:**
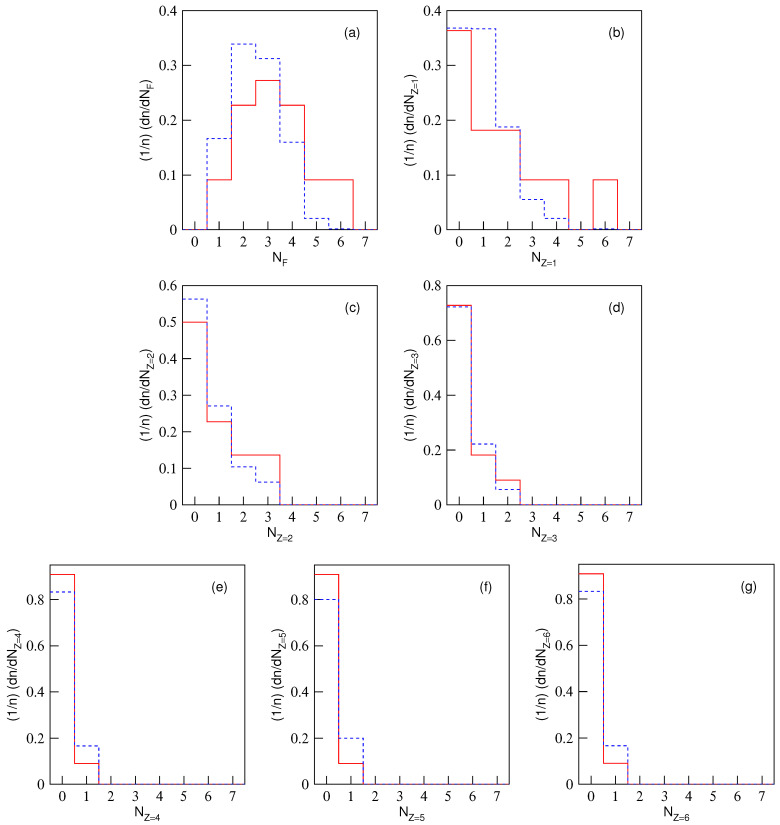
Multiplicity distributions of nuclear fragments with different charges in ^12^C fragmentation. The solid (dashed) histograms represent the results from the equal (unequal) probability partitioning method. Panel (**a**) is for all fragments. Panels (**b**–**g**) are for the fragments with charge Z=1, 2, ⋯, and 6, respectively.

**Figure 3:**
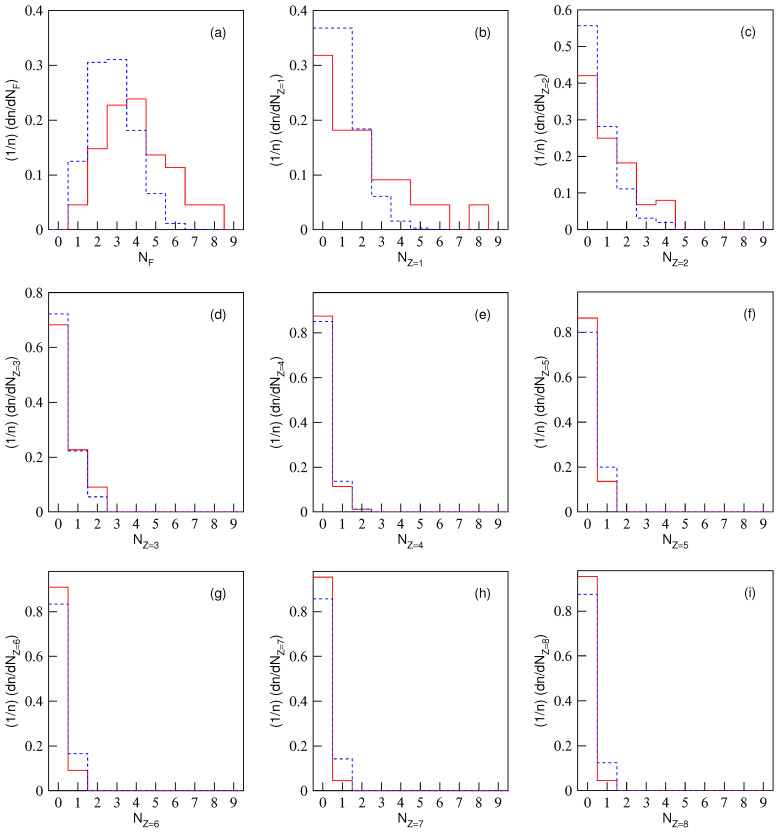
Multiplicity distributions of nuclear fragments with different charges in O16 fragmentation. The solid (dashed) histograms represent the results from the equal (unequal) probability partitioning method. Panel (**a**) is for all fragments. Panels (**b**–**i**) are for the fragments with charge Z=1, 2, ⋯, and 8, respectively.

The normalized multiplicity distributions, $\left(1/n\right)\left(dn/dN_{F}\right)$ or $\left(1/n\right)\left(dn/dN_{Z}\right)$, in excited Be9, C12, and O16 fragmentation are displayed in Figure 1, Figure 2 and Figure 3, respectively. The solid (dashed) histograms represent the results from the equal (unequal) probability partitioning method. Figure 1a, Figure 2a and Figure 3a are for the multiplicity distributions of all fragments. The multiplicity distributions of the fragments with different *Z* are shown in different panels, where Z=1–4 in Figure 1b–e, Z=1–6 in Figure 2b–g, and Z=1–8 in Figure 3b–i are for excited Be9, C12, and O16 fragmentation, respectively. It is worth noting that the predicted frequency distributions in Figure 1, Figure 2 and Figure 3 are precise numerical values (fractions) in each bin under a given scenario.

One can see from Table 4, Table 5 and Table 6 and Figure 1, Figure 2 and Figure 3 that the multiplicity distributions of the fragments with given *Z* from both the equal and unequal probability partitioning methods have a quick decreasing trend in most cases. The larger the *Z*, the closer the trends of the two results are. In the equal probability partitioning method, the priority of the 2He channel in Be9 fragmentation is significant, and the priority of the 3He (4He) channel in C12 (O16) fragmentation is not significant. In the unequal probability partitioning method, the three cases do not show an obvious priority. Figure 1a, Figure 2a and Figure 3a demonstrate peaks around the intermediate multiplicity, which are naturally different from the multiplicity distribution of the fragments with a given *Z*.

In addition, the multiplicity distribution of the fragments with Z=2 can be seen clearly, particularly in the equal probability partitioning method: $(dn/dN_{Z=2})/5=1.5/5=30\%$ for 2He channel in excited Be9 fragmentation, $(dn/dN_{Z=2})/11=1.5/11=13.64\%$ for 3He channel in excited C12 fragmentation, and $(dn/dN_{Z=2})/22=1.75/22=7.95\%$ for 4He channel in excited O16 fragmentation. In the unequal probability partitioning method, the three values are 5/24≈20.8%, 45/720=6.25%, and 777/40,320 ≈ 1.93%.

One can see that the difference between the two percentages from the equal and unequal probability partitioning methods in Be9 fragmentation is not too large, and that in O16 fragmentation is quite large. If the α-cluster structure does exist in excited nuclei formed in eA collisions at the EIC, one should observe much more multi-He configuration than these percentages (probabilities), which can be obtained from Table 4, Table 5 and Table 6 (Figure 1, Figure 2 and Figure 3). Although there are some experimental reports on the α-cluster structure of an excited nucleus [13,35,36,37,38], the related percentage or fraction of multi-He configurations is significantly smaller than that obtained through partitioning methods due to events with multi-particle production included in the data sample [35,36,37,38]. Thus, this fraction cannot be directly compared with partitioning results.

An experimental study using nuclear emulsion [57] found that the H + 2He channel fraction in B10 fragmentation at a beam energy of $E_{\mathrm{beam}}=1$ GeV/nucleon is 78%. Based on this finding, we estimate that the 2He channel fraction in Be9 fragmentation at $E_{\mathrm{beam}}=1$ GeV/nucleon is approximately 78%, possibly slightly higher due to fewer fragmentation channels for Be9 compared to B10. The inferred 2He channel fraction in excited Be9 fragmentation is estimated to be 2.6–3.8 times that from partitioning methods. As a non-conservative estimation, we set our judgment line for α clustering cases at twice the baseline percentages (probabilities) without α clustering.

In this context, we assume that the experimental percentage of multi-He events follows a Gaussian distribution with standard deviation σ, predominantly concentrated within the range [0,4σ] and centered around an expected value of 2σ, which serves as our baseline. If the experimental percentage exceeds 4σ, defined as twice the baseline and serving as our threshold for judgment, one can draw conclusions regarding the existence of He3 or α clustering with a confidence level over 95%. According to this line, we conclude that He3 or α clustering exists in excited Be9 formed during peripheral collisions between B10 and nuclear emulsion at $E_{\mathrm{beam}}=1$ GeV/nucleon.

Experimental data on the fragmentation of C9, C10, and C11 at an energy of $E_{\mathrm{beam}}=1.2$ GeV/ nucleon within nuclear emulsion show fractions for the 3He channel as follows: 15.2%, 5.3%, and 17.5%, respectively [58,59,60]. Additionally, experiments on O16 fragmentation at $E_{\mathrm{beam}}=3.65$ and 200 GeV/nucleon within nuclear emulsion indicate fractions for the 4He channel as 12.5% and 2.3%, respectively [61]. The fraction of multi-He channels in the fragmentation of excited C9,11 (O16) formed at $E_{\mathrm{beam}}=1.2$ (3.65) GeV/nucleon is more than double that predicted by unequal probability partitioning, suggesting the presence of He3 or α clustering in these excited nuclei. However, for excited C10 formed at $E_{\mathrm{beam}}=1.2$ GeV/nucleon and excited O16 formed at 200 GeV/nucleon, the multi-He channel fractions do not exceed twice those from unequal probability partitioning, indicating a stochastic result rather than He3 or α clustering.

It should be noted that the errors in experimental data quoted here are not available in refs. [57,58,59,60,61]. According to the errors in data for other channels [57], the relative errors for the quoted data are estimated by us to be 15–21%. Generally, at $E_{\mathrm{beam}}=1.2$ GeV/nucleon, the fraction of the 3He channel in excited C10 fragmentation is significantly lower than that in excited C9,11 due to its even-even nature, which enhances its stability and reduces He3 or α clustering probabilities while increasing other fragmentation channels’ likelihoods. Additionally, original α clustering presented in excited O16 at 3.65 GeV/nucleon is disrupted by violent collisions at 200 GeV/nucleon. These collisions lead to multi-particle production and participant nucleons separating from O16, making conditions for forming 4He clusters less favorable. Furthermore, higher excitation energy achieved with increased beam energy likely surpasses the threshold energies needed for forming such clusters; thus, higher-excited O16 fragments into multiple nucleons instead of favoring a 4He channel.

Based on the judgment line, the fractions of 2He (3He or 4He) channel in excited Be9 (C12 or O16) fragmentation should be higher than 60% (27.28% or 15.9%) if the equal probability partitioning method is considered, or 41.6% (12.5% or 3.86%) if the unequal probability partitioning method is considered. Here, these percentages are obtained from twice the values shown in Figure 1c, Figure 2c and Figure 3c, respectively, according to the assumption of twice the baselines. One may note that excited Be9 shows a significant 2He frequency and excited C12 (O16) does not show obvious enhancement of 3He (4He). The reason is that Be9 has very few fragmentation channels in total, and C12 (O16) has relatively more fragmentation channels in total. The tables and figures presented in the present work can be regarded as a benchmark reference result in which the α-cluster model does not enter. We look forward to the results of excited nuclear fragmentation at the forthcoming EIC experiments to study the fraction of multi-He configuration.

In addition, in the excited nucleus formed in eA collisions, a liquid–gas phase transition may also occur. In the above discussions on nuclear fragmentation, the liquid–gas phase transition is not taken into account in the calculations. If the liquid–gas phase transition occurs, more light fragments should be produced, causing the distribution of light fragment multiplicity to deviate from the histogram in Figure 1, Figure 2 and Figure 3, reducing the probability of low-multiplicity events and increasing the probability of high-multiplicity events. Meanwhile, heavy fragments should not be produced, or their yield should be very low. The results of this work can also provide reference for whether liquid–gas phase transition occurs in the excited nucleus in eA collisions at the future EIC.

In eA collisions, if the incident nucleus *A* is very large, the liquid-gas phase transition can occur in a part of the excited nucleus. For the local area, where the phase transition has occurred, many light fragments are expected to be emitted, and there is no intermediate and heavy fragment emitted with them. For the remainder area, where the phase transition has not happened, the fragmentation is not special, in which the multiplicity distribution of nuclear fragments should generally obey the partitioning methods.

To ensure as accurate a description as possible, the heaviest fragments produced in an event—considered remnants of excited nuclear fragmentation—should be excluded from analysis. For genuine evaporation products, it must be acknowledged that they originate from the fragmentation process involving a smaller excited nucleus; thus, the partitioning methods should be reapplied specifically for this smaller nucleus. Whether a liquid–gas phase transition occurs in the overall or local area, the proportion of light fragments with Z=1 should exceed twice the baseline values when applying Gaussian distribution to the considered probabilities. Furthermore, if experimental measurements fall within the theoretical uncertainty range, fragmentation may be interpreted as a consequence of a general stochastic process.

Considering that excited nuclei undergo liquid–gas phase transitions locally or globally, we take the probability of the fragmentation channels including 2H–4H (2H or 4H) in Be9, 3H–6H (3H, 4H, or 6H) in C12, and 4H–8H (4H, 5H, 6H, or 8H) in O16, from Table 1, Table 2 and Table 3, as the baseline values. Based on the baseline values, the fractions of channels including 2H–4H (3H–6H or 4H–8H) in excited Be9 (C12 or O16) fragmentation should be higher than 80% (54.55% or 45.45%) if the equal probability partitioning method is considered, or 58.33% (15.56% or 3.82%) if the unequal probability partitioning method is considered. Here, these percentages are derived by doubling the baseline values. The baseline values are assumed to be primarily concentrated within the range [0,4σ] when a Gaussian probability distribution with width σ is applied.

Beyond α clustering and liquid–gas phase transitions—which may lead to significant deviations between experimental multiplicity distributions and theoretical models—other nuclear effects exert only minor influences on experimental outcomes. These nuclear effects include non-uniform nucleon number density distributions (the neutron skin structure of heavy nuclei), symmetrical energy characteristics of nuclear matter, two- or multi-nucleon correlations within nuclei, as well as stopping power or transparency phenomena associated with nuclear interactions. Here, both the effects themselves and their impact can be neglected in studying the multiplicity distribution of nuclear fragments.

The reason why other nuclear effects are small is that they mainly affect the momentum distribution of nucleons inside the nucleus. Due to limited strength, the other nuclear effects mentioned above are not sufficient to affect the formation of nuclear fragments with given charge *Z*, though they affect the neutron numbers in emitted isotopes. As a result, they also affect the kinetic energy and emitting direction of nuclear fragments. In short, the transverse momenta and polar angles of nuclear fragments are significantly affected, while the charges and multiplicity of nuclear fragments are slightly affected.

## 4. Nonextensive Parameters from Multiplicity Distributions of Nuclear Fragments

Tsallis statistics represent an extension and generalization of Boltzmann–Gibbs statistics. They introduce the nonextensive entropy index *q*, built upon the foundational concept of Boltzmann–Gibbs statistics—the Boltzmann–Gibbs entropy—to construct a new form of entropy, known as the Tsallis (nonextensive or non-additive) entropy $S_{q}$ [62,63,64,65,66]. The Tsallis nonextensive statistical framework extends traditional Boltzmann–Gibbs statistics to describe systems characterized by long-range interactions, non-equilibrium dynamics, or fractal-like structures. In the limit q→1, both the entropy $S_{q}$ and the distribution function of Tsallis statistics reduce to their Boltzmann–Gibbs counterparts. Thus, Boltzmann–Gibbs statistics can be regarded as a special case of Tsallis statistics when q=1.

The probability density function used in Tsallis statistics has different forms or revisions [62,63,64,65,66]. For the multiplicity *N* distribution of nuclear fragments, we use

(7) $$\begin{array}{c} P\left(N\right)=P\left(0\right){\left[1-\frac{(1-q)N}{T_{q}}\right]}^{\frac{1}{1-q}}, \end{array}$$

where P(0) denotes the probability of events with zero multiplicity for fragments of a given charge number; $T_{q}$ is a generalized temperature that measures the average energy per degree of freedom in the generalized equilibrium state, distinct from the conventional temperature in thermal equilibrium systems; and *q* denotes entropy index which quantifies the degree of non-extensivity of the system. A value of q=1 corresponds to the Boltzmann–Gibbs limit, while q>1 indicates enhanced nonextensive behavior due to strong correlations or non-equilibrium effects.

In the description of the multiplicity distribution of nuclear fragments, the form of nonextensive or non-additive *q*-entropy $S_{q}$ is written by [62,63,64,65,66]

(8) $$\begin{array}{c} S_{q}=k\frac{1-\sum_{N}P_{N}^{q}}{q-1}, \end{array}$$

where *k* is the Boltzmann constant, which is equal to 1 in the natural units; and $P_{N}$ [=P(N)] denotes the probability of the nuclear fragment with multiplicity *N* and satisfying $\sum_{N}P_{N}=1$. $S_{q}$ measures the degree of disorder or complexity in the system, accounting for non-additive contributions from correlated subsystems.

**Figure 4:**
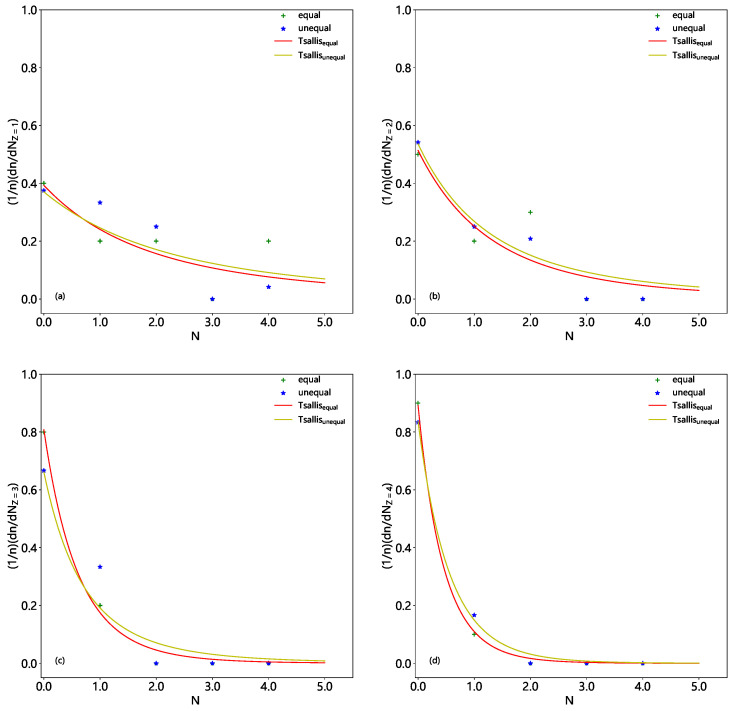
Multiplicity distributions of nuclear fragments with Z=1 (**a**), 2 (**b**), 3 (**c**), and 4 (**d**) in Be9 fragmentation. The crosses (asterisks) represent the results from the equal (unequal) probability partitioning method, which are cited from the solid (dashed) histograms in Figure 1. The corresponding results fitted by the Tsallis probability density function (Equation (7)) are presented by the red (yellow) curves.

**Figure 5:**
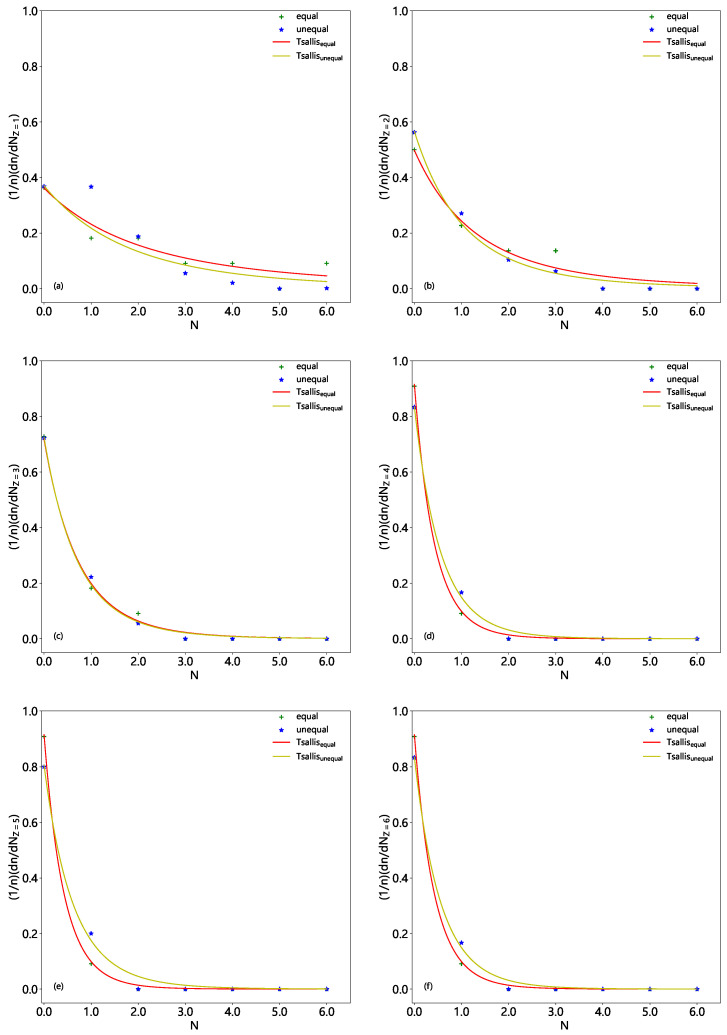
Multiplicity distributions of nuclear fragments with Z=1 (**a**), 2 (**b**), 3 (**c**), 4 (**d**), 5 (**e**), and 6 (**f**) in C12 fragmentation. The crosses (asterisks) represent the results from the equal (unequal) probability partitioning method, which are cited from the solid (dashed) histograms in Figure 2. The corresponding results fitted by the Tsallis probability density function (Equation (7)) are presented by the red (yellow) curves.

**Figure 6:**
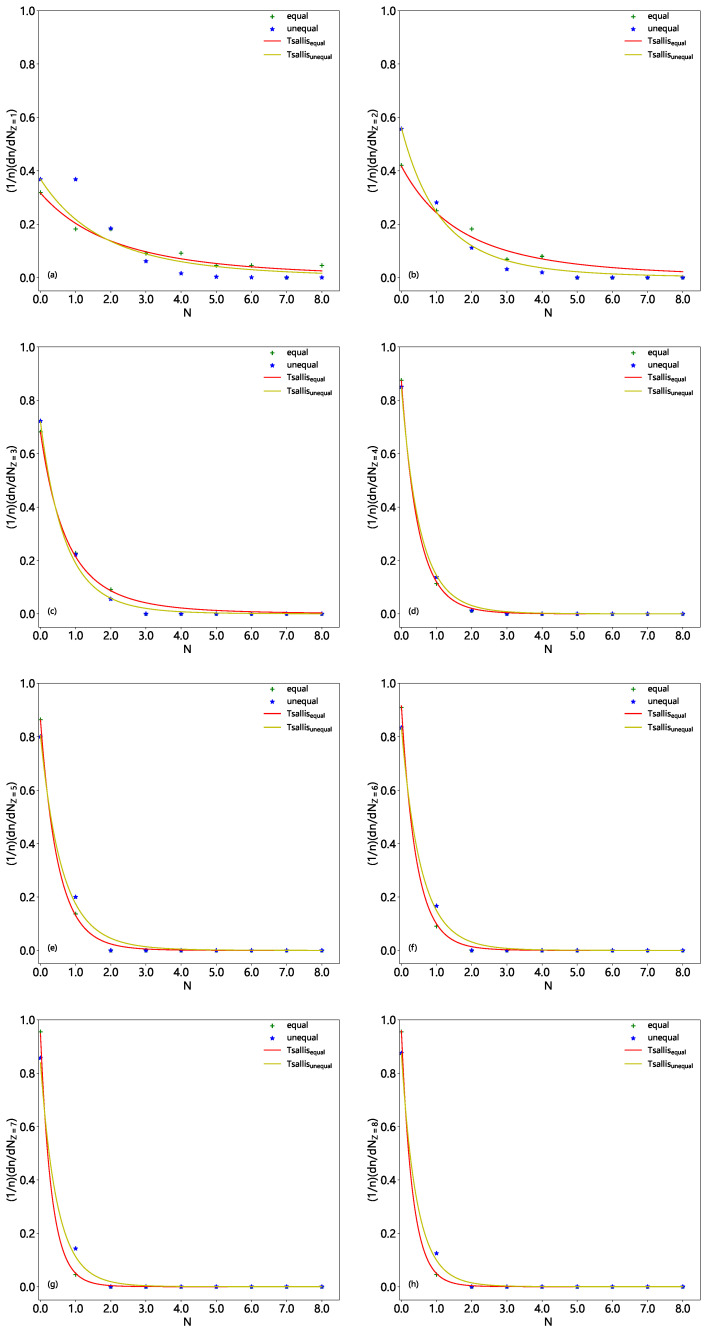
Multiplicity distributions of nuclear fragments with Z=1 (**a**), 2 (**b**), 3 (**c**), 4 (**d**), 5 (**e**), 6 (**f**), 7 (**g**), and 8 (**h**) in O16 fragmentation. The crosses (asterisks) represent the results from the equal (unequal) probability partitioning method, which are cited from the solid (dashed) histograms in Figure 3. The corresponding results fitted by the Tsallis probability density function (Equation (7)) are presented by the red (yellow) curves.

We present the multiplicity distributions of nuclear fragments with Z=1–4 (a–d), Z=1–6 (a–f), and Z=1–8 (a–h) in Be9, C12, and O16 fragmentations in Figure 4, Figure 5 and Figure 6, respectively. The crosses (asterisks) represent the results from the equal (unequal) probability partitioning method, which are cited from the solid (dashed) histograms in Figure 1, Figure 2 and Figure 3, respectively. The corresponding results fitted by the Tsallis probability density function (Equation (7)) are presented by the red (yellow) curves. When judging the goodness of fit, the coefficient of determination $R^{2}=1-\left(\mathrm{RSS}/\mathrm{TSS}\right)$ is used, where $\mathrm{RSS}=\sum {\left(y_{i}-{\hat{y}}_{i}\right)}^{2}$ represents the residual sum of squares, $\mathrm{TSS}=\sum {\left(y_{i}-\bar{y}\right)}^{2}$ denotes the total sum of squares, $y_{i}$ is the actual observed value of the *i*-th data point, ${\hat{y}}_{i}$ is the model-predicted value corresponding to the *i*-th data point, and $\bar{y}$ is the mean of the observed values. The values of $T_{q}$, *q*, and $R^{2}$ derived from Figure 4, Figure 5 and Figure 6 are summarized in Table 7. The closer $R^{2}$ is to 1, the better the model’s fit. One can see that in most cases, the Tsallis probability density function can approximately fit the multiplicity distribution of nuclear fragments with different charge numbers.

**Figure 7:**
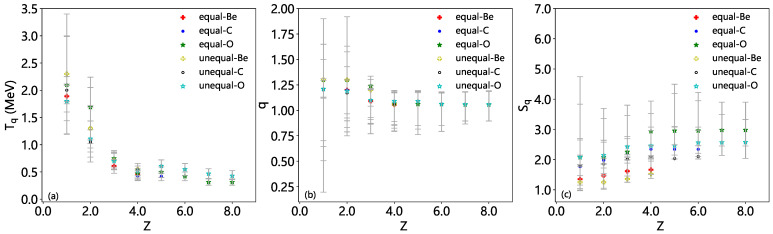
Dependencies of the Tsallis nonextensive parameters—(**a**) generalized temperature $T_{q}$, (**b**) entropy index *q*, and (**c**) *q*-entropy $S_{q}$—on fragment charge number *Z* in nuclear fragmentation reactions of Be9 (crosses), C12 (circles), and O16 (asterisks), determined via equal-probability (filled symbols) and unequal-probability (open symbols) partitioning methods.

The dependencies of Tsallis nonextensive parameters—including (a) $T_{q}$, (b) *q*, and (c) $S_{q}$—on the fragment charge number *Z* for nuclear fragmentation reactions of Be9 (crosses), C12 (circles), and O16 (asterisks) are shown in Figure 7, where $T_{q}$ and *q* listed in Table 7 are extracted from the fit of multiplicity distribution via Equation (7) and $S_{q}$ is obtained due to Equation (8). These results are derived using two distinct partitioning approaches: the equal-probability method (represented by filled symbols) and the unequal-probability method (represented by open symbols). The error bars in the free parameter figures are obtained by the $\chi^{2}$ profile method with a 95% confidence level. One can see the tendencies of the considered nonextensive parameters.

The generalized temperature $T_{q}$ exhibits a decreasing trend with increasing fragment charge number *Z* for all three excited nuclei (Be9, C12, and O16). This observation suggests that heavier fragments, which carry a larger proportion of the parent nucleus’s charge, are associated with lower effective temperatures. Physically, this can be interpreted as a result of the more ordered internal structure and lower excitation energy of heavier fragments, as they tend to retain more of the parent nucleus’s initial stability. Furthermore, a systematic independence of the excited nucleus mass is observed: the $T_{q}$ values (∼0.5–2 MeV) from the multiplicity distribution of O16 fragments are consistently in agreement with those from multiplicity distributions of C12 and Be9 fragments at the same *Z* due to all three nuclei being light. It is expected that heavier excited nuclei produce fragments with more stable configurations and lower average excitation energies, likely due to their higher binding energy per nucleon and more favorable fragmentation pathways, which minimize the release of excess energy. Notably, the difference between the equal-probability and unequal-probability partitioning methods is not significant in the whole *Z* region.

The values (∼1.05–1.3) of entropy index *q* are found to remain nearly unchanged within the uncertainty range, with increasing *Z* across the three excited nuclei. Similar *q* values support the possibility that collective excitation and surface effects may play a dominant role in high-energy fragmentation of light nuclei, rather than bulk behavior. The values of *q* deviate from 1 for all fragments, confirming the strong nonextensive nature of nuclear fragmentation reactions. This deviation from the Boltzmann–Gibbs limit (q=1) is attributed to the long-range nucleon–nucleon interactions, non-equilibrium dynamics during the fragmentation process, and the fractal-like structure of the phase space accessible to the fragments. Considering the uncertainty range, *q* has a probability of being smaller than 1, which indicates that the nuclear fragmentation system tends to suppress high-energy excited states, causing the particle distribution to be more concentrated in low-energy regions, which may occur in some confined or strongly dissipative systems, although this is less common in nuclear physics.

The *q*-entropy $S_{q}$ also remains almost within the uncertainty range, with the fragment charge number *Z* for all three excited nuclei. This indicates that the microstructural diversity or information uncertainty levels of these three types of light nucleus fragmentation final states are similar, meaning that the way and degree of system “chaos” are statistically equivalent, despite differences in fragment *Z* distribution. These three types of light nuclei exhibit statistical self-similarity during fragmentation, suggesting that one can use a unified and effective theoretical model (such as Tsallis statistics) to describe the dynamics of light nucleus fragmentation, without the need to fit parameters separately for each nucleus. The difference between the two partitioning methods can be neglected for the *q*-entropy $S_{q}$, even at large *Z* values. This indicates that the overall degree of disorder in the system is not as sensitive to the choice of partitioning method as the generalized temperature $T_{q}$ and the entropy index *q*. However, the unequal-probability method generally yields slightly smoother $S_{q}\left(Z\right)$ curves, as it accounts for the non-uniform probabilities of different fragmentation channels.

As mentioned above, the equal-probability partitioning method assumes that all possible fragmentation channels are equally likely, providing a simplified approach to parameter extraction. However, this assumption may not hold in reality, as certain fragmentation pathways may be favored due to quantum mechanical effects (e.g., shell structure) or energetic considerations (e.g., minimum energy configurations). In contrast, the unequal-probability partitioning method incorporates dynamical weights based on the physical likelihood of each fragmentation channel, offering a more realistic description of the reaction mechanism. As a result, this method generally leads to more reasonable $T_{q}$ and *q* values (when accounting for preferential pathways). The discrepancy between the two methods serves as a valuable indicator of the reliability of the extracted parameters. In regions where the differences are significant, it highlights the need for careful consideration of the underlying fragmentation dynamics and the choice of statistical framework.

Our analysis of the dependencies of Tsallis nonextensive parameters on the fragment charge number *Z* provides valuable insights into the statistical nature of nuclear fragmentation reactions. The observed trends in $T_{q}$, *q*, and $S_{q}$ with *Z* and excited nucleus mass show that nuclear fragmentation reactions result in a generalized equilibrium state that deviates significantly from the traditional thermal equilibrium, as evidenced by the nonextensive parameter values (q≠1). Heavier fragments exhibit lower effective temperatures of emission source, but similar nonextensive behavior and similar nonextensive entropy compared to lighter fragments. These similarities reflect the consistency in statistical behavior and dynamic evolution, and the universal mechanism of the fragmentation process.

The choice of partitioning method (equal-probability vs. unequal-probability) has no notable impact on the extracted parameters, particularly for the generalized temperature $T_{q}$ and the entropy index *q*. However, the unequal-probability method, which accounts for the physical likelihood of different fragmentation channels, should provide a more accurate and self-consistent description of the reaction dynamics. Our findings underscore the utility of the Tsallis nonextensive statistical framework in characterizing complex nuclear reactions and highlight the importance of considering non-equilibrium effects and correlated dynamics in such systems.

Before the Section 5, we would like to emphasize that while Tsallis statistics demonstrate strong applicability in fitting fragment multiplicity distributions, the underlying physical mechanisms merit further exploration. A core feature of Tsallis statistics is their nonextensivity, characterized by the parameter *q*, which allows them to describe systems with long-range interactions or significant fluctuations. During fragment production, the complexity of the collision process and diversity of intermediate states give rise to substantial system fluctuations that cannot be captured by traditional equilibrium statistics. Tsallis nonextensive statistics, however, can precisely account for distribution deviations caused by such fluctuations, enabling successful fitting of multiplicity distributions.

In addition, Tsallis statistics can effectively describe particle production behavior during the formation of quark-gluon plasma (QGP). Fragment production and QGP formation may share key similarities—both involve strong interactions and particle cascade processes in non-equilibrium states, which could be one potential reason for the applicability of Tsallis statistics in nuclear fragmentation. Our research reveals that the nearly invariant trend of parameter *q* within the uncertainty range as *Z* changes is closely linked to energy dissipation and particle correlation during fragmentation: as *Z* increases, the degree to which *q* deviates from 1 does not change, indicating similar local fluctuations within the system. This finding further validates the use of Tsallis statistics for describing non-equilibrium fragment production processes.

## 5. Summary and Conclusions

Various configurations of nuclear fragments resulting from the fragmentation of excited Be9, C12, and O16 nuclei—expected to form in eA collisions at the EIC—are investigated using both equal- and unequal-probability partitioning methods. The multiplicity distributions for all fragments, as well as those with charge *Z*, are derived. In comparison to results obtained from these partitioning methods, experiments suggest that multi-α configurations should exhibit a significantly high probability according to the α-cluster model. We anticipate that the structure of excited nuclei featuring an α-cluster will be clearly manifested and further validated in future studies. According to our predictions, the fraction of the 2He (3He or 4He) channel in excited Be9 (C12 or O16) fragmentation should exceed 60% (27.28%, 15.9%) under the equal-probability partitioning method, or 41.6% (12.5%, 3.86%) under the unequal-probability partitioning method, with over 95% confidence level.

Additionally, findings from this work can serve as a reference for assessing whether a liquid-gas phase transition occurs within excited nucleus in eA collisions. Should such a phase transition take place experimentally, an increased observation of light fragments is expected alongside minimal detection of heavy fragments. For very heavy excited nuclei, it is plausible that liquid–gas phase transition could occur in a specific region where numerous light fragments are evaporated while another area undergoes a fragmentation process or has a remaining smaller nucleus; this fragmentation process may deviate from traditional partitioning methods if significant α-clustering is present. We predict that the fraction of channels spanning 2H–4H (3H–6H or 4H–8H) in excited Be9 (C12 or O16) fragmentation should exceed 80% (54.55% or 45.45%) under the equal-probability partitioning method, or 58.33% (15.56% or 3.82%) under the unequal-probability partitioning method, with a condifence level over 95%.

In the framework of Tsallis statistics, the nonextensive parameters $T_{q}$, *q*, and $S_{q}$ are obtained from the multiplicity distribution of nuclear fragments with a given *Z*. With the increase of *Z*, $T_{q}$ decreases, while both *q* and $S_{q}$ remain nearly unchanged within the uncertainty range. Our work shows that fragmentation of nuclear remnants in electron–nucleus collisions at high energy is a nonextensive process (q≈1.05–1.3) with a temperature of $T_{q}\approx 0.5$–2 MeV. This work reveals the transformative utility of the Tsallis nonextensive statistical framework in decoding the previously uncharacterized complexities of nuclear fragmentation reactions, challenging traditional equilibrium-based models and emphasizing the urgent need to integrate non-equilibrium effects and correlated dynamics into the core of nuclear reaction theory.

Before the end of this paper, we would like to point out that our study relies solely on the α-cluster model and two partitioning methods (equal and unequal probability). However, the actual fragmentation process in eA collisions likely involves more complex correlated dynamics and non-equilibrium effects not captured by these models. The Tsallis statistics, while revealing nonextensive features, remain a phenomenological approach lacking a microscopic foundation for the fragmentation mechanism. The predicted fragmentation channel fractions (multi-He fragment dominance) and liquid–gas phase transition signatures (multi-H fragment dominance) require future experimental verification. Moreover, we have only examined three not-too-heavy excited nuclei; the fragmentation behavior of heavier nuclei (e.g., medium and heavy mass) under eA collisions remains unexplored, limiting the generalizability of our conclusions.

Future work should develop a microscopic model that incorporates α-clustering and non-equilibrium dynamics into the fragmentation process, going beyond phenomenological Tsallis statistics. This could involve coupling the α-cluster model with dynamical equations to describe the time evolution of excited nuclei. Modelers should collaborate with experimental groups at the EIC to design dedicated measurements of α-cluster configurations and light fragment multiplicities, and to develop advanced data analysis techniques for improved detection efficiency and fragment identification. The theoretical framework should also be extended to medium- and heavy-mass nuclei, investigating the liquid–gas phase transition in these systems and testing the universality of fragmentation patterns observed in light nuclei. Finally, a microscopic derivation of the Tsallis nonextensive statistical parameters would help establish a more fundamental connection between the fragmentation process and the underlying nuclear dynamics.

## Figures and Tables

**Table 1:** The multiplicity, $N_{F}$, of all fragments and the multiplicity, $N_{Z}$, of the fragments with charge *Z* in various configurations in excited Be9 fragmentation, where only the charge conservation is considered in the fragmentation. In the equal probability partitioning method, the fragment Be is defaulted with 50% probability to be Be8, and in the unequal probability partitioning method, the fragment Be is defaulted with a given chance $\left\{M_{2}\left(2\mathrm{He}\right)/\left[M_{2}\left(2\mathrm{He}\right)+M_{2}\left(\mathrm{Be}\right)\right]=1/3\right\}$ to be Be8, where Be8 is unstable and can decay into 2He, which is listed in the bracket, and causes $N_{F}$ to $N_{F}+1$ and $N_{Z=2}$ to $N_{Z=2}+2$. Here, the changeable $N_{F}$ and $N_{Z=2}$ are shown in the table by +1 and +2, respectively. The probabilities $f_{1}$ and $f_{2}$ of each channel obtained by the equal and unequal partitioning methods are listed respectively.

$N_{F}$	$N_{Z=1}$	$N_{Z=2}$	$N_{Z=3}$	$N_{Z=4}$	Configuration	$f_{1}$ (1/5)	$f_{2}$ (1/24)
4	4				4H	1	1
3	2	1			2H + He	1	6
2	1		1		H + Li	1	8
2		2			2He	1	3
1				1	[Be	0.5	4
1 + 1		+2			(2He)]	0.5	2

**Table 2:** The multiplicity $N_{F}$ of all fragments and the multiplicity $N_{Z}$ of the fragments with charge *Z* in various configurations in excited C12 fragmentation, where only the charge conservation is considered in the fragmentation. In the equal probability partitioning method, the fragment Be is defaulted with 50% probability to be Be8, and in the unequal probability partitioning method, the fragment Be is defaulted with a given chance (1/3) to be Be8, where Be8 is unstable and can decay into 2He, which is listed in the bracket, and causes $N_{F}$ to $N_{F}+1$ and $N_{Z=2}$ to $N_{Z=2}+2$. The probabilities $f_{1}$ and $f_{2}$ of each channel obtained by the equal and unequal partitioning methods are listed respectively.

$N_{F}$	$N_{Z=1}$	$N_{Z=2}$	$N_{Z=3}$	$N_{Z=4}$	$N_{Z=5}$	$N_{Z=6}$	Configuration	$f_{1}$ (1/11)	$f_{2}$ (1/720)
6	6						6H	1	1
5	4	1					4H + He	1	15
4	3		1				3H + Li	1	40
4	2	2					2H + 2He	1	45
3	2			1			[2H + Be	0.5	60
3 + 1	2	+2					2H + (2He)]	0.5	30
3	1	1	1				H + He + Li	1	120
3		3					3He	1	15
2	1				1		H + B	1	144
2		1		1			[He + Be	0.5	60
2 + 1		1 + 2					He + (2He)]	0.5	30
2			2				2Li	1	40
1						1	C	1	120

**Table 3:** The multiplicity $N_{F}$ of all fragments and the multiplicity $N_{Z}$ of the fragments with charge *Z* in various configurations in excited O16 fragmentation, where only the charge conservation is considered in the fragmentation. In the equal probability partitioning method, the fragment Be is defaulted with 50% probability to be Be8, and in the unequal probability partitioning method, the fragment Be is defaulted with a given chance (1/3) to be Be8, where Be8 is unstable and can decay into 2He, which is listed in the bracket, and causes $N_{F}$ to $N_{F}+1$ and $N_{Z=2}$ to $N_{Z=2}+2$. The probabilities $f_{1}$ and $f_{2}$ of each channel obtained by the equal and unequal partitioning methods are listed respectively.

$N_{F}$	$N_{Z=1}$	$N_{Z=2}$	$N_{Z=3}$	$N_{Z=4}$	$N_{Z=5}$	$N_{Z=6}$	$N_{Z=7}$	$N_{Z=8}$	Configuration	$f_{1}$ (1/22)	$f_{2}$ (1/40,320)
8	8								8H	1	1
7	6	1							6H + He	1	28
6	5		1						5H + Li	1	112
6	4	2							4H + 2He	1	210
5	4			1					[4H + Be	0.5	280
5 + 1	4	+2							4H + (2He)]	0.5	140
5	3	1	1						3H + He + Li	1	1120
5	2	3							2H + 3He	1	420
4	3				1				3H + B	1	1344
4	2	1		1					[2H + He + Be	0.5	1680
4 + 1	2	1 + 2							2H + He + (2He)]	0.5	840
4	2		2						2H + 2Li	1	1120
4	1	2	1						H + 2He + Li	1	1680
4		4							4He	1	105
3	2					1			2H + C	1	3360
3	1	1			1				H + He + B	1	4032
3	1		1	1					[H + Li + Be	0.5	2240
3 + 1	1	+2	1						H + (2He) + Li]	0.5	1120
3		2		1					[2He + Be	0.5	840
3		2 + 2							2He + (2He)]	0.5	420
3		1	2						He + 2Li	1	1120
2	1						1		H + N	1	5760
2		1				1			He + C	1	3360
2			1		1				Li + B	1	2688
2				2					[2Be	0.25	504
2 + 1		+2		1					(2He) + Be	0.5	504
2 + 1 + 1		+2 + 2							(2He) + (2He)]	0.25	252
1								1	O	1	5040

**Table 4:** The multiplicity distribution, $dn/dN_{F}$, of all fragments and the multiplicity distribution, $dn/dN_{Z}$, of the fragments with charge *Z* in excited Be9 fragmentation in the equal (unequal) probability partitioning method, where the normalization is 5 (24), which is the number of total configurations (exchanges). In the table, $N_{x}$ denotes $N_{F}$ or $N_{Z}$.

$\mathrm{dn}/{\mathrm{dN}}_{x}$	$N_{x}=0$	$N_{x}=1$	$N_{x}=2$	$N_{x}=3$	$N_{x}=4$
$dn/dN_{F}$	0 (0)	0.5 (4)	2.5 (13)	1 (6)	1 (1)
$dn/dN_{Z=1}$	2 (9)	1 (8)	1 (6)	0 (0)	1 (1)
$dn/dN_{Z=2}$	2.5 (13)	1 (6)	1.5 (5)	0 (0)	0 (0)
$dn/dN_{Z=3}$	4 (16)	1 (8)	0 (0)	0 (0)	0 (0)
$dn/dN_{Z=4}$	4.5 (20)	0.5 (4)	0 (0)	0 (0)	0 (0)

**Table 5:** The multiplicity distribution $dn/dN_{F}$ of all fragments and the multiplicity distribution $dn/dN_{Z}$ of the fragments with charge *Z* in excited C12 fragmentation in the equal (unequal) probability partitioning method, where the normalization is 11 (720), which is the number of total configurations (exchanges).

$\mathrm{dn}/{\mathrm{dN}}_{x}$	$N_{x}=0$	$N_{x}=1$	$N_{x}=2$	$N_{x}=3$	$N_{x}=4$	$N_{x}=5$	$N_{x}=6$
$dn/dN_{F}$	0 (0)	1 (120)	2.5 (244)	3 (225)	2.5 (115)	1 (15)	1 (1)
$dn/dN_{Z=1}$	4 (265)	2 (264)	2 (135)	1 (40)	1 (15)	0 (0)	1 (1)
$dn/dN_{Z=2}$	5.5 (405)	2.5 (195)	1.5 (75)	1.5 (45)	0 (0)	0 (0)	0 (0)
$dn/dN_{Z=3}$	8 (520)	2 (160)	1 (40)	0 (0)	0 (0)	0 (0)	0 (0)
$dn/dN_{Z=4}$	10 (600)	1 (120)	0 (0)	0 (0)	0 (0)	0 (0)	0 (0)
$dn/dN_{Z=5}$	10 (576)	1 (144)	0 (0)	0 (0)	0 (0)	0 (0)	0 (0)
$dn/dN_{Z=6}$	10 (600)	1 (120)	0 (0)	0 (0)	0 (0)	0 (0)	0 (0)

**Table 6:** The multiplicity distribution $dn/dN_{F}$ of all fragments and the multiplicity distribution $dn/dN_{Z}$ of the fragments with charge *Z* in excited O16 fragmentation in the equal (unequal) probability partitioning method, where the normalization is 22 (40,320), which is the number of configurations (exchanges).

$\mathrm{dn}/{\mathrm{dN}}_{x}$	$N_{x}=0$	$N_{x}=1$	$N_{x}=2$	$N_{x}=3$	$N_{x}=4$	$N_{x}=5$	$N_{x}=6$	$N_{x}=7$	$N_{x}=8$
$dn/dN_{F}$	0 (0)	1 (5040)	3.25 (12,312)	5 (12,516)	5.25 (7301)	3 (2660)	2.5 (462)	1 (28)	1 (1)
$dn/dN_{Z=1}$	7 (14,833)	4 (14,832)	4 (7420)	2 (2464)	2 (630)	1 (112)	1 (28)	0 (0)	1 (1)
$dn/dN_{Z=2}$	9.25 (22,449)	5.5 (11,340)	4 (4494)	1.5 (1260)	1.75 (777)	0 (0)	0 (0)	0 (0)	0 (0)
$dn/dN_{Z=3}$	15 (29,120)	5 (8960)	2 (2240)	0 (0)	0 (0)	0 (0)	0 (0)	0 (0)	0 (0)
$dn/dN_{Z=4}$	19.25 (34,272)	2.5 (5544)	0.25 (504)	0 (0)	0 (0)	0 (0)	0 (0)	0 (0)	0 (0)
$dn/dN_{Z=5}$	19 (32,256)	3 (8064)	0 (0)	0 (0)	0 (0)	0 (0)	0 (0)	0 (0)	0 (0)
$dn/dN_{Z=6}$	20 (33,600)	2 (6720)	0 (0)	0 (0)	0 (0)	0 (0)	0 (0)	0 (0)	0 (0)
$dn/dN_{Z=7}$	21 (34,560)	1 (5760)	0 (0)	0 (0)	0 (0)	0 (0)	0 (0)	0 (0)	0 (0)
$dn/dN_{Z=8}$	21 (35,280)	1 (5040)	0 (0)	0 (0)	0 (0)	0 (0)	0 (0)	0 (0)	0 (0)

**Table 7:** Values of $T_{q}$, *q*, and $R^{2}$ used for the curves in Figure 4, Figure 5 and Figure 6 under equal and unequal probability partitioning methods. Due to retaining three decimal places and rounding the fourth decimal place, some $R^{2}$ values are 1.000.

Figure		Equal			Unequal	
**Number**	$T_{q}$ **(MeV)**	q	$R^{2}$	$T_{q}$ **(MeV)**	q	$R^{2}$
Figure 4a	$1.890_{-0.450}^{+0.225}$	$1.300_{-1.104}^{+0.200}$	0.620	$2.300_{-0.550}^{+0.685}$	$1.300_{-0.265}^{+0.349}$	0.728
Figure 4b	$1.300_{-0.210}^{+0.750}$	$1.200_{-0.276}^{+0.430}$	0.787	$1.300_{-0.435}^{+0.389}$	$1.300_{-0.460}^{+0.275}$	0.920
Figure 4c	$0.610_{-0.135}^{+0.116}$	$1.090_{-0.320}^{+0.119}$	0.994	$0.710_{-0.170}^{+0.151}$	$1.200_{-0.550}^{+0.102}$	0.926
Figure 4d	$0.450_{-0.096}^{+0.078}$	$1.055_{-0.230}^{+0.117}$	0.999	$0.550_{-0.550}^{+0.105}$	$1.062_{-0.795}^{+0.122}$	0.997
Figure 5a	$2.300_{-0.510}^{+1.101}$	$1.300_{-0.170}^{+0.600}$	0.885	$1.810_{-0.810}^{+0.250}$	$1.155_{-0.556}^{+0.095}$	0.825
Figure 5b	$1.300_{-0.281}^{+0.420}$	$1.200_{-0.235}^{+0.229}$	0.963	$1.050_{-0.278}^{+0.224}$	$1.170_{-0.273}^{+0.129}$	0.990
Figure 5c	$0.730_{-0.141}^{+0.161}$	$1.100_{-0.172}^{+0.141}$	0.996	$0.710_{-0.155}^{+0.145}$	$1.100_{-0.236}^{+0.115}$	0.997
Figure 5d	$0.420_{-0.082}^{+0.080}$	$1.060_{-0.210}^{+0.122}$	1.000	$0.550_{-0.115}^{+0.105}$	$1.062_{-0.269}^{+0.115}$	0.998
Figure 5e	$0.420_{-0.082}^{+0.080}$	$1.060_{-0.210}^{+0.122}$	1.000	$0.610_{-0.137}^{+0.112}$	$1.090_{-0.328}^{+0.104}$	0.994
Figure 5f	$0.420_{-0.082}^{+0.080}$	$1.060_{-0.210}^{+0.122}$	1.000	$0.550_{-0.115}^{+0.105}$	$1.062_{-0.269}^{+0.115}$	0.998
Figure 6a	$2.100_{-0.500}^{+0.090}$	$1.300_{-0.180}^{+0.600}$	0.944	$1.500_{-0.599}^{+0.290}$	$1.200_{-0.509}^{+0.100}$	0.846
Figure 6b	$1.690_{-0.750}^{+0.550}$	$1.300_{-0.512}^{+0.275}$	0.958	$1.110_{-0.427}^{+0.325}$	$1.190_{-0.358}^{+0.173}$	0.988
Figure 6c	$0.750_{-0.202}^{+0.137}$	$1.240_{-0.330}^{+0.095}$	0.995	$0.700_{-0.153}^{+0.135}$	$1.100_{-0.228}^{+0.101}$	0.997
Figure 6d	$0.470_{-0.095}^{+0.087}$	$1.066_{-0.176}^{+0.116}$	1.000	$0.520_{-0.115}^{+0.090}$	$1.090_{-0.230}^{+0.105}$	0.999
Figure 6e	$0.500_{-0.103}^{+0.092}$	$1.061_{-0.245}^{+0.112}$	0.999	$0.610_{-0.138}^{+0.109}$	$1.090_{-0.328}^{+0.098}$	0.995
Figure 6f	$0.420_{-0.082}^{+0.080}$	$1.060_{-0.210}^{+0.121}$	1.000	$0.550_{-0.115}^{+0.102}$	$1.062_{-0.268}^{+0.107}$	0.998
Figure 6g	$0.313_{-0.059}^{+0.060}$	$1.056_{-0.161}^{+0.128}$	1.000	$0.468_{-0.087}^{+0.092}$	$1.061_{-0.195}^{+0.118}$	0.998
Figure 6h	$0.313_{-0.059}^{+0.060}$	$1.056_{-0.161}^{+0.128}$	1.000	$0.430_{-0.072}^{+0.095}$	$1.060_{-0.165}^{+0.132}$	0.999

## Data Availability

The data used to support the findings of this study are included within the article and are cited at relevant places within the text as references.
